# Effects of Exercise Training and Weight Loss on Plasma Fetuin-A Levels and Insulin Sensitivity in Overweight Older Men

**DOI:** 10.1155/2017/1492581

**Published:** 2017-07-09

**Authors:** Jacob B. Blumenthal, Anna Gitterman, Alice S. Ryan, Steven J. Prior

**Affiliations:** ^1^Division of Gerontology and Geriatric Medicine, Department of Medicine, University of Maryland School of Medicine, Baltimore, MD, USA; ^2^Baltimore Veterans Affairs Geriatric Research, Education and Clinical Center and Research and Development Service, Baltimore, MD, USA

## Abstract

Aerobic exercise training and weight loss (AEX+WL) improves insulin sensitivity in overweight adults; however, the underlying pathways are incompletely understood. Fetuin-A, a hepatokine that inhibits insulin signaling, may be involved in the salutary effects of AEX+WL. Therefore, we examined the effects of 6-month AEX+WL on plasma fetuin-A levels (36–48 hours after the last bout of exercise), aerobic capacity (VO_2max_), body composition, glucose tolerance, and insulin sensitivity (M) in 16 sedentary, overweight-obese older men (age = 60 ± 2 years, BMI = 31 ± 1 kg/m^2^) with no history of cardiovascular disease or diabetes. At baseline, fetuin-A levels correlated directly with adiposity and had a borderline inverse correlation with M. After AEX+WL, body weight decreased by ~10 kg, while both VO_2max_ and M increased by 16% (*P* < 0.005 for all). Contrary to our hypothesis, plasma fetuin-A levels increased after AEX+WL (1.16 ± 0.10 g/L versus 1.70 ± 0.19 g/L, *P* = 0.006). This increase was unrelated to changes in body composition or glucose metabolism, but directly correlated with changes in VO_2max_ (*r* = 0.57, *P* < 0.05). Thus, in overweight-to-obese older men, AEX+WL appears to increase plasma fetuin-A levels. Although not associated with improvements in insulin sensitivity, this increase in fetuin-A was related to improvements in aerobic capacity and could be representative of the cardioprotective effects of AEX+WL in older men.

## 1. Introduction

Obesity is a growing problem in the United States where approximately two-thirds of adults aged 20 years or older are overweight or obese (BMI ≥ 25) [1]. Linked to increased weight is metabolic dysfunction (e.g., type 2 diabetes and dyslipidemia) that may, in part, be attributable to cytokines and hepatokines such as fetuin-A (*α*2-Heremans-Schmid glycoprotein). Fetuin-A is an abundant serum protein, almost exclusively expressed and secreted by hepatocytes and adipocytes, whose levels are upregulated in hepatic steatosis and metabolic disorders [2, 3]. In particular, fetuin-A has been shown to bind the insulin receptor and inhibit autophosphorylation of tyrosine kinase to decrease insulin signaling in cell culture models [4, 5]. Fetuin-A also has been implicated in promoting insulin resistance through proinflammatory effects in human monocytes and adipocytes, as well as reducing expression of adiponectin in human adipocytes in vitro [6]. In a number of cross-sectional and observational studies in humans, fetuin-A has been linked to insulin resistance and diabetes, often independently of body composition and other cardiovascular disease risk factors [2, 7–9]. Similarly, a relationship between circulating fetuin-A levels and CVD risk has been demonstrated in older adults [9, 10]; however, it is noteworthy that there is a direct relationship between fetuin-A levels and CVD risk in individuals with type 2 diabetes, while this relationship is inverse in older adults without type 2 diabetes [9].

Subcutaneous and visceral adipose tissue secrete fetuin-A, and in animal models, circulating levels of fetuin-A vary directly with percent fat mass when fat mass is manipulated by experimental conditions such as diet-induced obesity, exercise training, and weight loss induced by activity-based anorexia [11]. Furthermore, weight loss has been demonstrated to reduce circulating fetuin-A levels in humans [12, 13]. To date, few studies have addressed the effects of aerobic exercise training (AEX) with or without weight loss (WL) on fetuin-A and its relationship to metabolic outcomes, and the results of these studies have been somewhat contradictory. Six weeks of AEX with modest changes in body composition had no effect on serum fetuin-A levels in obese older women [14]; however, 12 weeks of AEX with significant WL did reduce plasma fetuin-A levels in a study of obese older men and women [15]. It is unclear whether these discrepant findings are attributable to differences in the duration of the intervention, weight loss, or the sex of the participants; however, there is some evidence that relationships between fetuin-A levels and metabolic outcomes differ between men and women [16].

To deepen our understanding of the relationship between fetuin-A and cardiometabolic outcomes, we examined the effects of longer duration AEX+WL in a group of overweight-to-obese, older men, without the potential confounding effect of sex differences. We tested the hypothesis that six-month AEX+WL would reduce circulating fetuin-A levels and that this reduction would be associated with improvements in insulin sensitivity.

## 2. Materials and Methods

Sedentary, overweight, or obese men 50–75 years of age without a history of either cardiovascular disease or diabetes were recruited from the greater Baltimore-Washington D.C. region for participation in exercise and weight loss studies. Data from sixteen men are reported herein; subject characteristics and certain metabolic data from nine of these subjects were previously reported as part of another study [17]. Participants were screened by medical history, physical examination, and treadmill exercise tests. Exclusion criteria included (i) cancer, as well as hematological, pulmonary, renal, or thyroid disease; (ii) medications such as beta-blockers, steroids, statins, or medications normally prescribed for diabetes; and (iii) poorly controlled hypertension, dyslipidemia, or anemia. All subjects were nonsmokers, sedentary (no moderate or vigorous exercise >20 minutes on 2 or more days/week), and weight stable (no recent weight change of more than 2 kg) and reported consuming no more than two alcoholic drinks per day. All study procedures were approved by the Institutional Review Board at the University of Maryland School of Medicine, and all participants provided written informed consent.

### 2.1. Study Protocol

Prior to research testing, all subjects received instruction on maintaining a weight-stable, therapeutic lifestyle changes diet by a registered dietitian 1 day per week for 6–8 weeks. All subjects were weight-stable (±2%) for at least 2 weeks prior to research testing and were provided an isocaloric diet for 2 days before testing to control nutrient intake, as previously described [18]. Subjects were also asked to refrain from any moderate-to-vigorous physical exercise during this 2-day period. Similar conditions were maintained for postintervention testing; however, testing was performed 36–48 hours after the last bout of exercise.

#### 2.1.1. Maximal Oxygen Consumption (VO_2max_)

VO_2max_ was measured using indirect calorimetry (Quark, Cosmed, Chicago, IL, USA) during a graded exercise test on a motorized treadmill as previously described [18, 19]. Briefly, subjects walked at a constant velocity throughout the protocol; grade was initially set to 0% and increased every 2 minutes thereafter to maximal effort. VO_2max_ was defined as the highest oxygen consumption value obtained for a full 30-second increment. All subjects attained VO_2max_ as evidenced by standard physiological criteria (respiratory exchange ratio > 1.10 or a plateau in VO_2_ with an increase in workload).

#### 2.1.2. Body Mass Index and Body Composition

Body weight was measured to the nearest 0.1 kg with an electronic scale, and standing height was measured to the nearest 0.1 cm using a wall-mounted stadiometer. Body mass index (BMI) was calculated by dividing body weight (kilograms) by height (meters squared). Body composition (fat mass, lean mass, and percent body fat) was determined by dual-energy X-ray absorptiometry (Prodigy, Lunar Radiation Corp., Madison, WI, USA).

#### 2.1.3. Oral Glucose Tolerance Test

All subjects underwent a 2-hour oral glucose tolerance test (OGTT) after a 12-hour overnight fast, as previously described [18]. A catheter was placed in an antecubital vein, and blood samples were drawn before and every 30 minutes after the ingestion of a 75 g glucose solution for 2 hours. Blood samples were centrifuged, and plasma was separated and stored at −80°C until analysis. Plasma glucose levels were analyzed with a glucose analyzer (YSI 2300 STAT Plus, YSI, Yellow Springs, OH, USA). Glucose area under the curve (G_AUC_) was calculated using the trapezoidal method.

#### 2.1.4. Hyperinsulinemic-Euglycemic Clamp

Insulin-stimulated glucose uptake (M) was measured as an index of insulin sensitivity. Subjects were provided with all meals for the 2 days preceding the clamp to control nutrient intake. After a 12-hour overnight fast, subjects underwent hyperinsulinemic-euglycemic clamp [20, 21] as performed in our laboratory [22]. Insulin was infused at a rate of 80 mU/m^2^/min, and M is reported in mg of glucose infused per kilogram of body weight per minute (mg/kg/min) or micromoles of glucose infused per kilogram of fat-free mass per minute (*μ*mol/kgFFM/min). Plasma glucose levels were analyzed at 5-minute intervals using the glucose oxidase method (Beckman Instruments, Fullerton, CA). Plasma insulin levels were determined by radioimmunoassay (EMD Millipore, St. Charles, MO).

#### 2.1.5. Plasma Fetuin-A Levels

Fetuin-A concentration in plasma was measured by enzyme-linked immunosorbent assays (ELISA) (Epitope Diagnostics, San Diego, CA) according to the manufacturer's instructions. Each sample was analyzed in triplicate, with the mean of three values used for statistical analyses; the intra-assay coefficient of variation was less than 8%. All samples from each subject (before and after AEX+WL) were analyzed on the same plate.

#### 2.1.6. AEX+WL

After the completion of baseline testing, subjects underwent 6 months of AEX training at the Baltimore VA GRECC exercise facility supervised by trained exercise physiologists. AEX training began at a volume of 3 sessions/week of 20 minutes at 50% of VO_2max_ and gradually increased to 3 sessions/week of 45 minutes at 60–70% of VO_2max_, a level maintained for >4 months. During this 6-month period, subjects also met weekly with a registered dietician and were counseled to restrict their caloric intake by 500 kcal/day to achieve >5% weight loss. At the end of the 6-month intervention, the testing battery was repeated under identical conditions.

#### 2.1.7. Statistical Analysis

Data are presented as means ± SEM. All statistical analyses were performed using IBM SPSS v22 (IBM, Armonk, NY, USA). Paired *t*-tests were used to test for differences in outcome variables before and after AEX+WL. Bivariate correlation analyses and regression analyses were used to test for associations between fetuin-A levels and metabolic variables. A type I error rate of *α* = 0.05 was selected, and two-tailed probabilities are reported for all analyses.

## 3. Results

Sixteen participants (mean age = 60 ± 2 years, BMI = 31 ± 1 kg/m^2^) completed the six-month AEX+WL intervention, and their metabolic data are shown in Table 1. At baseline, fetuin-A levels directly correlated with percent fat (*r* = 0.59, *P* = 0.016) and with BMI (*r* = 0.43, *P* = 0.01) and tended to correlate inversely with M expressed in mL/kg/min (*r* = −0.45, *P* = 0.08), but not with M expressed in *μ*mol/kgFFM/min (*r* = −0.35, *P* = 0.18). Fetuin-A levels did not correlate with measures of glucose tolerance (fasting glucose, 120 minute glucose, or G_AUC_), nor with VO_2max_.

The AEX+WL intervention resulted in significant 10% losses in weight (−9.5 ± 1.7 kg, *P* < 0.001) and body fat (−4.5 ± 0.8%, *P* < 0.001), as well as a 16% improvement in VO_2max_ (+0.40 ± 0.11 L/min, *P* = 0.003). These changes in body composition and aerobic capacity were accompanied by improvements in cardiometabolic measures, including 6% decreases in systolic and diastolic blood pressure (*P* < 0.05), a 12% increase in high-density lipoprotein cholesterol (HDL-C; *P* < 0.05), an 8% decrease in G_AUC_, and a 16% increase in M (*μ*mol/kgFFM/min, *P* < 0.001).

**Figure 1:**
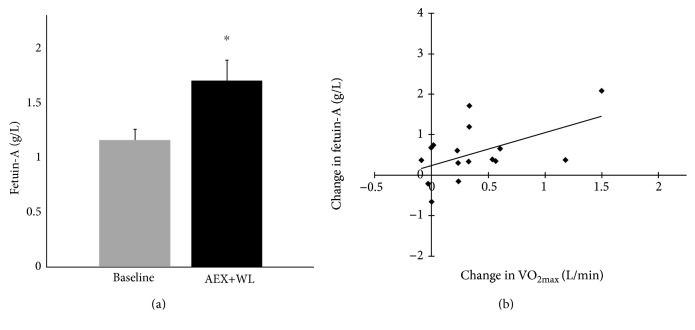
(a) Plasma fetuin-A levels at baseline and after aerobic exercise training and weight loss (AEX+WL). ^∗^Significant difference compared with baseline, *P* = 0.006. (b) Scatterplot depicting the relationship between AEX+WL-induced changes in VO_2max_ and circulating fetuin-A levels. The changes in fetuin-A level directly correlated with changes in VO_2max_ (*r* = 0.57, *P* = 0.027).

Contrary to our hypothesis, fetuin-A levels increased 47% after AEX+WL (Figure 1(a), *P* = 0.006). In addition, despite the correlation between percent fat and fetuin-A at baseline, the changes in fetuin-A level were unrelated to changes in weight or body composition (*r* = 0.06–0.14, *P* > 0.63). The AEX+WL induced increases in fetuin-A level correlated only with those in aerobic capacity expressed as VO_2max_ in L/min (Figure 1(b), *r* = 0.57, *P* = 0.027), and neither with changes in M nor 120-minute postprandial glucose (*r* = −0.26  to  − 0.37, *P* > 0.18). Inclusion of the change in weight or fat mass did not affect the association of the changes in fetuin-A levels with VO_2max_ (VO_2max_ partial *r* = 0.59, *P* = 0.036).

## 4. Discussion

Circulating levels of fetuin-A previously have been associated with diabetes risk and hypothesized to play a role in the development of insulin resistance in overweight and obese people. Therefore, we assessed the effects of six-month AEX+WL on circulating fetuin-A levels as a potential mediator of AEX+WL-induced improvements in insulin sensitivity and cardiometabolic outcomes. As expected, participants lost weight and increased VO_2max_, while also realizing improvements in insulin sensitivity, blood pressure, and lipoprotein-lipid profiles after AEX+WL. However, contrary to our hypothesis, plasma fetuin-A levels increased significantly following AEX+WL, in direct correlation with the improvements in VO_2max_. This increase in fetuin-A was unrelated to the improvement in insulin sensitivity, a finding inconsistent with a direct contribution of fetuin-A to improvements in glucose metabolism after AEX+WL in older men.

In rodent models, fetuin-A has been shown to bind the insulin receptor where it inhibits the autophosphorylation of tyrosine kinase to decrease insulin signaling [4], and in murine skeletal muscle, fetuin-A may similarly inhibit AS160 phosphorylation [23]. Fetuin-A also has been implicated to promote insulin resistance through proinflammatory effects, including augmenting cytokine expression and reducing that of adiponectin [6]. Despite subsequent findings of associations between fetuin-A levels, insulin resistance, and the development of diabetes in humans [7, 24], few studies have assessed the effects of AEX or WL interventions on circulating fetuin-A levels and relationships to metabolic outcomes. In morbidly obese women, bariatric surgery resulting in 35% weight loss reduced fetuin-A levels, which correlated with improvements in glucose tolerance [12]. Another study reported more modest decreases (<5%) in fetuin-A levels after dietary WL [13]. However, given the differences in WL and lack of AEX intervention, it is difficult to directly compare these findings with the present study. In one study of WL with increases in physical activity, fetuin-A levels decreased in a subset of participants, but this appeared dependent on a reduction in liver fat content [25]. In another study of obese older men and women, 12 weeks of AEX and a degree of WL similar to the present study significantly reduced plasma fetuin-A levels [15]. This reduction in fetuin-A levels was associated with both body composition and metabolic improvements; however, similar to the present report, the change in fetuin-A levels did not correlate with the change in insulin-stimulated glucose uptake during a glucose clamp [15]. Recently, Lee et al. have reported that circulating levels of fetuin-A decreased ~11% in middle-aged men in response to a 12-week program of combined endurance and strength training; these changes in circulating fetuin-A interacted with those in free fatty acids to predict some of the improvements in insulin sensitivity [26]. Other studies have reported equivocal results in response to diet and exercise interventions. Schultes et al. found no change in serum fetuin-A levels in obese, premenopausal women after a 6-week AEX intervention that induced only modest (<1%) changes in body fat [14]. Similarly, Yang et al. found no change in serum fetuin-A levels in middle-aged women after a three-month AEX and strength training intervention that resulted in ~5% WL [27]. Given the discrepant findings among studies of different sex and age groups, different methods of WL (with or without AEX), and different timing of fetuin-A measurement (e.g., with 24 hours versus 36–48 hours after the last bout of AEX in the present study), a more comprehensive study of changes in fetuin-A levels as a function of lifestyle interventions appears necessary.

It also remains possible that the observed association between fetuin-A levels and insulin resistance is not causal in humans. For example, a large study assessing variation in the gene encoding fetuin-A found that common genetic variants were strongly associated with plasma fetuin-A level, but there was no relationship with risk for type 2 diabetes or plasma glucose levels [28]. Our finding of improvements in insulin sensitivity despite increases in plasma fetuin-A levels is inconsistent with fetuin-A playing a major causal role in insulin resistance in humans.

Nonetheless, while we did not find associations with insulin sensitivity, it is possible that higher fetuin-A levels seen after AEX+WL could play a protective role in preventing ectopic calcifications, as improved vascular health and reduced cardiovascular disease risk are known benefits of exercise, and fetuin-A levels have been shown to be inversely associated with the presence of calcified plaques [29, 30]. In fact, fetuin-A is highly effective in the formation and stabilization of protein-mineral colloids and acts as a carrier for both calcium and phosphate [31]. With regard to this issue too, somewhat discordant results have previously been reported, with direct correlations between fetuin-A levels and carotid artery intima-media thickness, as well as inverse correlations with coronary artery calcification and global measures of atherosclerosis [30, 32–35]. Furthermore, although lower fetuin-A levels were associated with greater CVD risk in nondiabetic people, in diabetic people, the opposite trend was observed [36]. Finally, a recent meta-analysis of 10 case-control studies including over 1200 patients with cardiovascular disease and 2600 healthy controls concluded that lower serum fetuin-A levels correlated with the development of cardiovascular disease [37]. Additional evidence suggests that there may be a sex-specific relationship between fetuin-A, VO_2max_, and coronary artery calcification. Wilund et al. [38] reported a strong, direct correlation between plasma fetuin-A level and VO_2max_, which correlated inversely with coronary artery calcification in older men but not in older women. In this context, the increase in fetuin-A observed in the present study, along with improvements in VO_2max_, HDL-C, and blood pressure, could be interpreted as overall beneficial to reduce CVD risk through AEX+WL in overweight-to-obese older men.

The limitations and strengths of the present study are worthy of note. First, the population studied was relatively homogeneous, being limited to overweight-obese older men, and only whole-body insulin sensitivity was measured. Fetuin-A could have different effects in women, in those with lower degrees of obesity, or on hepatic insulin sensitivity specifically. Similarly, because those with either documented cardiovascular disease or diabetes were excluded, the relationship of fetuin-A to the measured cardiometabolic variables may have been underestimated. Although the longitudinal design is a strength of this study, the lack of a control group is a limitation. Similarly, the lack of groups undergoing AEX or WL alone precludes our distinguishing between the specific effects of AEX or WL. Nonetheless, both careful characterization and the intensity of the intervention are strengths of the present study, and the almost 50% increase in fetuin-A minimizes the likelihood that the effect size was insufficient for the latter to be reflected in any of the metabolic or body composition variables measured. Finally, we are presently unable to identify specific mechanisms underlying the observed increases in fetuin-A levels in response to AEX+WL in older men. While changes in liver function, liver fat content, or secretion of fetuin-A by hepatocytes and adipocytes all have the potential to mitigate fetuin-A levels, future studies are needed to assess these specific variables and mechanisms that may explain our findings.

## 5. Conclusions

We report an increase in plasma fetuin-A levels following a 6-month AEX+WL protocol in overweight-to-obese older men that correlates with the increase in VO_2max_, although not with other observed improvements in insulin sensitivity. Thus, in overweight-obese older men, fetuin-A does not appear to play a major role in these metabolic improvements induced by AEX+WL, although the increase in fetuin-A could potentially confer benefit with regard to CVD risk.

## Figures and Tables

**Table 1:** Subject characteristics and responses to aerobic exercise training with weight loss (AEX+WL).

	Baseline	6-month AEX+WL	*P* value
Age	60 ± 2	—	—
Weight (kg)	99.0 ± 3.5	89.5 ± 3.2	**<0.001**
BMI (kg/m^2^)	30.7 ± 0.8	28.2 ± 0.8	**<0.001**
Body fat (%)	31.9 ± 1.0	27.4 ± 1.4	**<0.001**
Fat mass (kg)	31.7 ± 1.9	25.4 ± 2.1	**<0.001**
Fat-free mass (kg)	65.2 ± 2.0	64.2 ± 2.0	0.10
VO_2max_ (L/min)	2.46 ± 0.14	2.86 ± 0.17	**0.003**
Fasting plasma glucose (mmol/L)	5.27 ± 0.09	5.21 ± 0.14	0.60
2 hr postprandial glucose (mmol/L)	7.67 ± 0.57	6.63 ± 0.59	0.15
Glucose AUC (mmol/L/120 min)	976 ± 49	901 ± 51	**0.03**
M (mg/kg/min)	5.23 ± 0.46	6.77 ± 0.55	**<0.001**
M (*μ*mol/kgFFM/min)	43.8 ± 4.1	50.8 ± 3.8	**0.004**
Total cholesterol (mg/dL)	4.46 ± 0.22	4.27 ± 0.23	0.25
HDL-C (mg/dL)	0.99 ± 0.05	1.11 ± 0.06	**0.009**
LDL-C (mg/dL)	2.89 ± 0.18	2.67 ± 0.19	0.15
Triglycerides (mg/dL)	1.27 ± 0.11	1.15 ± 0.14	0.32
Systolic BP (mmHg)	122 ± 3	114 ± 2	**0.03**
Diastolic BP (mmHg)	75 ± 1	70 ± 2	**0.02**

Data are means ± SEM. BMI: body mass index; VO_2max_: maximal oxygen consumption; AUC: area under the curve; M: insulin-stimulated glucose uptake; FFM: fat-free mass; HDL-C: high-density lipoprotein cholesterol; LDL-C: low-density lipoprotein cholesterol; BP: blood pressure.
